# Total Pectoral Muscle Area and Prognostic Nutritional Index Provide Complementary Prognostic Information for Patients Hospitalized with HFmrEF/HFrEF

**DOI:** 10.3390/jcm15176712

**Published:** 2026-08-29

**Authors:** Çetin Alak, Hasan Emin Kaya, Fazıl Çağrı Hunutlu, Muhammed Batuhan Daştan, Zeynep Kumral, Tunay Şentürk

**Affiliations:** 1 Department of Cardiology, Faculty of Medicine, Bursa Uludag University, Bursa 16059, Türkiye; tunaysenturk@gmail.com; 2 Department of Radiology, Faculty of Medicine, Bursa Uludag University, Bursa 16059, Türkiyebatuhan.dastan@yahoo.com.tr (M.B.D.); 3 Division of Hematology, Department of Internal Medicine, Bursa Sehir Training and Research Hospital, University of Health Sciences, Bursa 16009, Türkiye; fazilhunutlu@gmail.com; 4 Department of Cardiology, Unye State Hospital, Ordu 52300, Türkiye; zeynepkumral@gmail.com

**Keywords:** heart failure, HFmrEF, HFrEF, thoracic computed tomography, pectoral muscle area, prognostic nutritional index, sarcopenia, risk stratification

## Abstract

**Background:** Skeletal muscle depletion and malnutrition are common in heart failure (HF) and are associated with poor outcomes. Whether imaging-derived skeletal muscle reserve and laboratory-based nutritional indices provide complementary prognostic information remains unclear. We evaluated the prognostic value of total pectoral muscle area (TPMA), Prognostic Nutritional Index (PNI), and Controlling Nutritional Status (CONUT) scores in patients hospitalized with HFmrEF/HFrEF. **Methods:** In this retrospective single-center study, consecutive patients hospitalized with decompensated HF between 2017 and 2025 who underwent thoracic computed tomography were included. TPMA was measured at the T4 level. Nutritional status was assessed using PNI and CONUT. Receiver operating characteristic analysis, Kaplan–Meier analysis, Spearman correlation, and Cox regression were used to evaluate long-term all-cause mortality. **Results:** A total of 138 patients (median age 66 years; 35.5% women) were included. During a median follow-up of 34.4 months, 87 patients (63.0%) died. Non-survivors had lower TPMA (2589 vs. 3012 mm^2^, *p* = 0.002) and PNI (46.0 ± 5.5 vs. 50.2 ± 7.2, *p* < 0.001). TPMA (AUC 0.661, *p* = 0.001) and PNI (AUC 0.663, *p* = 0.001), but not CONUT (AUC 0.561, *p* = 0.226), predicted mortality. TPMA correlated modestly with PNI (ρ = 0.325, *p* < 0.001). Low TPMA (<3000 mm^2^; HR 1.911, *p* = 0.018), higher BNP (HR 1.024 per 100 pg/mL, *p* = 0.014), and lower PNI (HR 0.956, *p* = 0.024) independently predicted mortality. **Conclusions:** Reduced TPMA and lower PNI were independently associated with long-term mortality in hospitalized patients with HFmrEF/HFrEF. Their modest correlation suggests that they may capture partly distinct aspects of systemic impairment; however, the incremental prognostic value of their combined assessment requires further evaluation.

## 1. Introduction

Heart failure (HF) remains a major cause of morbidity, mortality, and recurrent hospitalization worldwide despite substantial advances in pharmacological and device-based therapies [1]. Beyond impaired cardiac function, HF is increasingly recognized as a systemic syndrome characterized by chronic inflammation, neurohormonal activation, metabolic dysregulation, and progressive functional decline, all of which contribute to adverse clinical outcomes [2,3].

Skeletal muscle depletion is an important extracardiac manifestation of HF and a central component of HF-associated sarcopenia [3]. Persistent inflammation, neurohormonal and hormonal disturbances, anabolic resistance, reduced physical activity, impaired peripheral perfusion, and nutritional deficiencies promote progressive loss of skeletal muscle mass and function [2,3]. These alterations contribute to frailty, exercise intolerance, and reduced survival. In a recent meta-analysis including more than 36,000 patients with HF, low muscle mass was associated with a 73% higher risk of all-cause mortality, underscoring the prognostic relevance of body composition assessment in this population [4]. Reduced skeletal muscle mass has also been linked to adverse cardiac remodeling, higher natriuretic peptide concentrations, and worse clinical outcomes [5].

Although the clinical diagnosis of sarcopenia requires assessment of muscle strength, muscle quantity or quality, and physical performance, imaging-derived measurements provide an objective assessment of skeletal muscle quantity [6]. Computed tomography (CT) is particularly suitable for opportunistic body composition analysis because skeletal muscle can be quantified from examinations obtained for routine clinical indications without additional imaging, radiation exposure, or cost [3,7]. Thoracic CT-derived muscle measurements correlate with established body composition parameters [7] and have demonstrated prognostic value in HF populations [8,9,10]. Low skeletal muscle mass assessed by chest CT has been independently associated with mortality in acute HF, while reduced pectoralis muscle area has been linked to adverse outcomes in both acute and advanced HF cohorts [8,9,10]. Thus, thoracic CT-derived pectoral muscle measurements may serve as accessible imaging markers of skeletal muscle reserve rather than as stand-alone diagnostic measures of sarcopenia.

Nutritional impairment is another common but frequently underrecognized systemic manifestation of HF. Malnutrition may contribute to skeletal muscle loss through inadequate nutrient intake, impaired intestinal absorption, inflammation, and catabolic imbalance [3,11]. However, nutritional status and skeletal muscle quantity represent related but distinct biological domains. Several clinical screening tools, including the Nutritional Risk Screening 2002 (NRS-2002) and Mini Nutritional Assessment (MNA), have been used to identify nutritional risk in patients with HF. Importantly, their associations with clinical outcomes have not been uniform across studies, and sex-specific differences in the prognostic impact of nutritional impairment have also been reported [12,13]. The Prognostic Nutritional Index (PNI), calculated from serum albumin concentration and lymphocyte count, provides a laboratory-based estimate of nutritional and immunological status and has consistently demonstrated prognostic value in HF populations [14,15,16]. Similarly, the Controlling Nutritional Status (CONUT) score, which incorporates serum albumin, total cholesterol, and lymphocyte count, has been associated with adverse outcomes in HF and supported by recent meta-analyses [17,18]. Nevertheless, these indices do not directly quantify skeletal muscle reserve.

Although both skeletal muscle depletion and nutritional impairment have been associated with adverse outcomes in HF, they have largely been investigated separately. Consequently, the relationship between thoracic CT-derived pectoral muscle area and laboratory-based nutritional indices remains poorly characterized. It is also uncertain whether these measures reflect a shared underlying process or provide independent and complementary prognostic information in patients hospitalized with decompensated HF.

Therefore, the present study aimed to evaluate the associations of total pectoral muscle area (TPMA), PNI, and CONUT scores with long-term all-cause mortality and to determine the relationship between imaging-derived skeletal muscle reserve and laboratory-based nutritional status in patients hospitalized with decompensated HF and a left ventricular ejection fraction (LVEF) below 50%.

## 2. Materials and Methods

### 2.1. Study Design and Population

This retrospective, observational, single-center study was conducted at the Department of Cardiology, Bursa Uludağ University Faculty of Medicine. Consecutive adult patients hospitalized with decompensated HF between 1 January 2017 and 1 May 2025 were identified through the institutional electronic database using International Classification of Diseases (ICD) coding records. Given the retrospective study design, the diagnosis and assessment of HF were based on the ESC guidelines applicable at the time of the index hospitalization: the 2016 ESC Guidelines for the diagnosis and treatment of acute and chronic heart failure during the earlier study period and the 2021 ESC Guidelines following their publication and implementation [19,20]. OpenAI ChatGPT (GPT-5; OpenAI, San Francisco, CA, USA) was used solely for English translation and language editing to improve clarity, grammar, and manuscript organization. All scientific content, study design, data analysis, interpretation of the results, and final editorial decisions were performed and verified by the authors.

Patients were considered eligible if thoracic CT imaging had been performed during the index hospitalization and complete clinical, laboratory, echocardiographic, and follow-up data were available. Thoracic CT examinations were obtained as part of routine clinical care for various clinical indications, including the evaluation of respiratory symptoms, suspected pulmonary or pleural pathology, and abnormalities identified on chest radiography. CT imaging was not performed according to a predefined study protocol, and the specific indication for CT could not be systematically categorized for all patients because of the retrospective study design.

**Figure 1:**
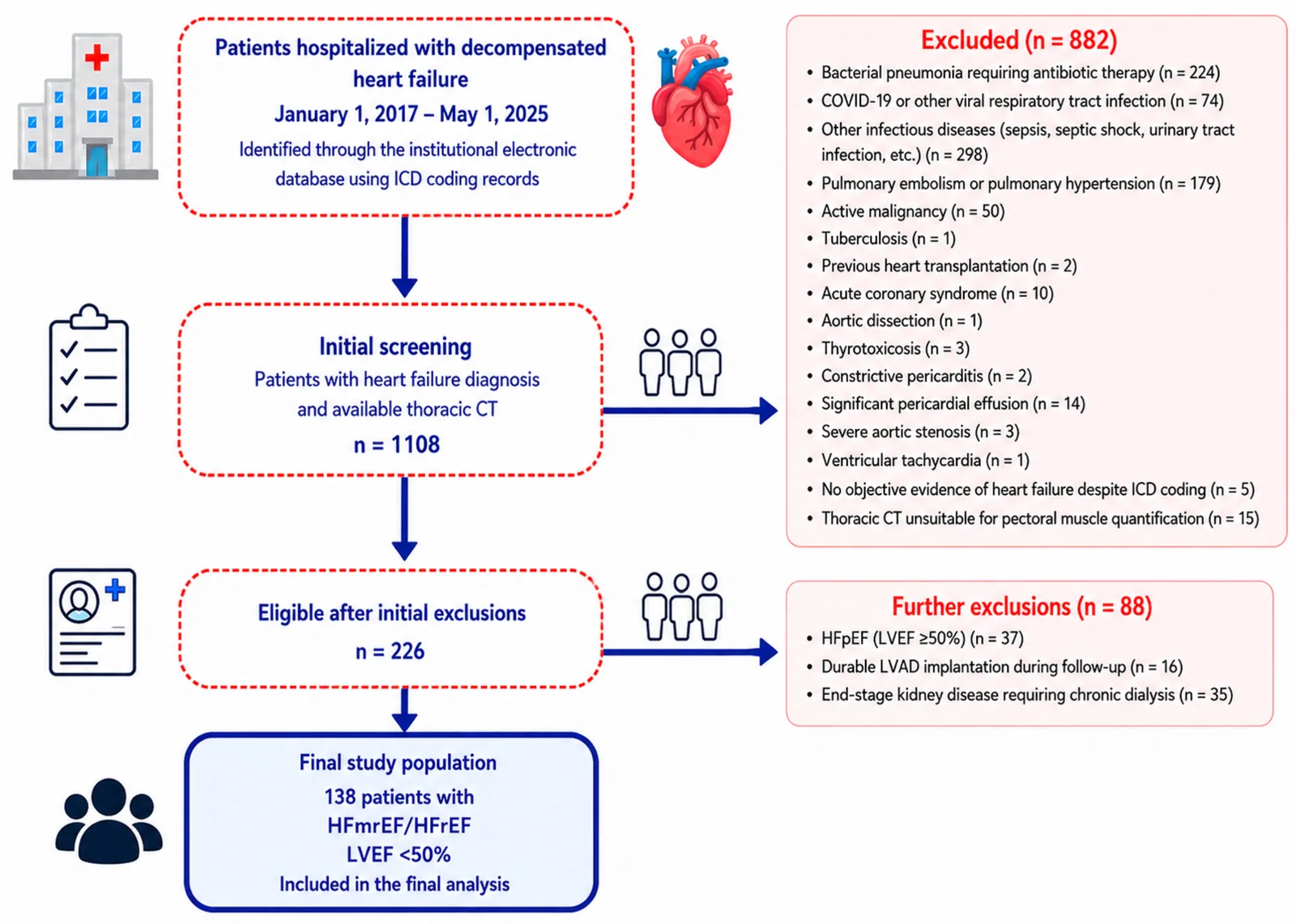
Study Flow Diagram.

A total of 1108 patients with a diagnosis of HF and available thoracic CT imaging were initially screened. Given the substantial influence of infection, inflammation, malignancy, and other catabolic conditions on both nutritional biomarkers and skeletal muscle measurements, patients with active infectious diseases, active malignancy, acute coronary syndromes, severe valvular disease, pulmonary vascular disease, prior heart transplantation, and other conditions potentially affecting body composition assessment were excluded. Detailed exclusion criteria are presented in Figure 1.

To obtain a clinically homogeneous cohort, patients with heart failure with preserved ejection fraction (HFpEF; LVEF ≥ 50%), patients who underwent durable left ventricular assist device (LVAD) implantation during follow-up, and patients with end-stage kidney disease requiring chronic dialysis were further excluded. The final study population consisted of 138 patients with heart failure and mildly reduced or reduced ejection fraction (HFmrEF/HFrEF), defined as an LVEF < 50%.

### 2.2. Clinical and Laboratory Data Collection

Demographic characteristics, cardiovascular risk factors, comorbid conditions, heart failure pharmacotherapy, laboratory findings, hospitalization records, and follow-up information were retrospectively retrieved from the institutional electronic medical record system. Heart failure pharmacotherapy documented during index hospitalization was recorded and included angiotensin-converting enzyme inhibitors (ACEis), angiotensin receptor blockers (ARBs), angiotensin receptor–neprilysin inhibitors (ARNIs), beta-blockers, mineralocorticoid receptor antagonists (MRAs), and sodium–glucose cotransporter 2 (SGLT2) inhibitors.

Baseline laboratory measurements obtained during the index hospitalization were used for analysis. Collected laboratory variables included hemoglobin, platelet count, serum creatinine, estimated glomerular filtration rate (eGFR), serum sodium, serum potassium, serum chloride, albumin, lymphocyte count, total cholesterol, low-density lipoprotein cholesterol, high-density lipoprotein cholesterol, triglycerides, B-type natriuretic peptide (BNP), ferritin, serum iron, transferrin saturation (TSAT), and C-reactive protein (CRP). BNP was the natriuretic peptide assay routinely used at our institution throughout the entire study period (2017–2025) and was available for all patients included in the analysis. Biochemical measurements, including CRP, albumin, and total cholesterol, were performed using an ARCHITECT c16000 automated clinical chemistry analyzer (Abbott Laboratories, Abbott Park, IL, USA). Conventional CRP was measured using the standard, non-high-sensitivity application of the MULTIGENT CRP Vario latex immunoturbidimetric assay (Abbott Laboratories, Abbott Park, IL, USA); hs-CRP was not used. Complete blood count parameters, including lymphocyte count, were measured using CELL-DYN Ruby and Alinity hematology analyzers (Abbott Laboratories, Abbott Park, IL, USA) during the study period.

The primary study endpoint was all-cause mortality. Survival status was determined using hospital records and the national death notification system. Follow-up duration was calculated from the date of index hospitalization until death or the last documented follow-up visit.

### 2.3. Thoracic Computed Tomography Acquisition and Body Composition Analysis

All chest CT examinations were performed as part of routine clinical care using either a 32-slice multidetector CT scanner (SOMATOM Perspective, Siemens Healthcare, Erlangen, Germany) or a 160-slice multidetector CT scanner (Aquilion Serve, Canon Medical Systems, Otawara, Japan).

Images obtained using the SOMATOM Perspective scanner were acquired at 130 kVp with a single collimation width of 1.2 mm, total collimation width of 19.2 mm, spiral pitch factor of 0.6, and reconstructed slice thickness of 1.5 mm. Images obtained using the Aquilion Serve scanner were acquired at 120 kVp with a single collimation width of 0.5 mm, total collimation width of 40 mm, spiral pitch factor of 0.813, and reconstructed slice thickness of 1.0 mm.

**Figure 2:**
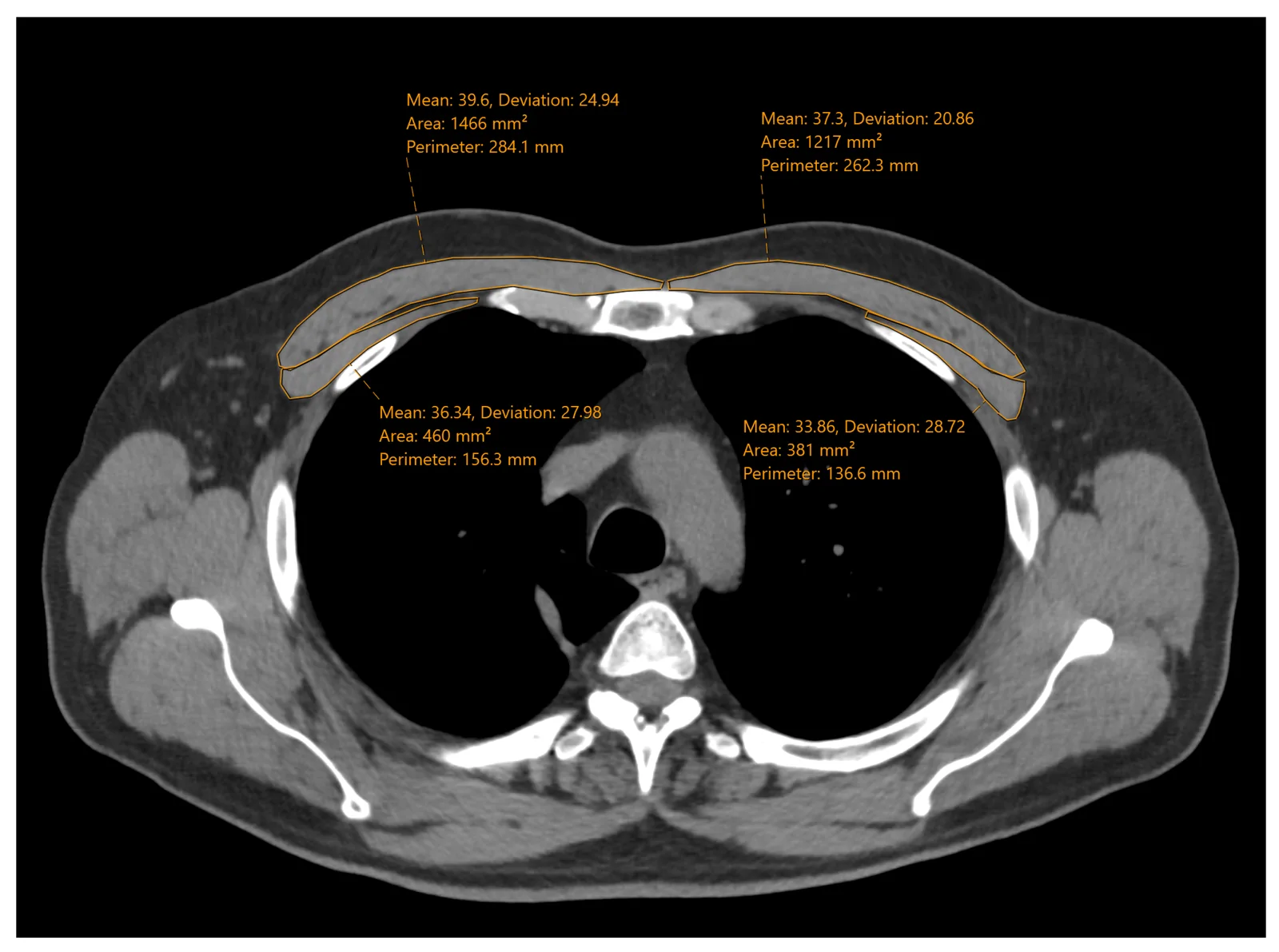
Representative axial thoracic computed tomography image illustrating measurement of the cross-sectional areas of the bilateral pectoralis major and minor muscles at the T4 level. The total pectoral muscle area (TPMA) was calculated as the sum of the measured muscle areas.

For body composition analysis, skeletal muscle area was assessed at the level of the fourth thoracic vertebra (T4), a validated thoracic landmark for muscle quantification. The left and right pectoralis major and minor muscles were manually delineated on a single axial image at the T4 vertebral level, consistent with previously described CT-based approaches to thoracic skeletal muscle quantification [21,22]. The cross-sectional areas of all four muscle components were summed to obtain the total pectoral muscle area (TPMA), expressed in mm^2^. A representative axial thoracic CT image illustrating the measurement of the bilateral pectoralis major and minor muscle areas at the T4 level is shown in Figure 2.

All image analyses were performed retrospectively on archived Digital Imaging and Communications in Medicine (DICOM) datasets by experienced radiologists who were blinded to clinical characteristics and outcome data.

### 2.4. Nutritional Assessment

Nutritional status was evaluated using the PNI and the CONUT score.

PNI was calculated according to the original Onodera formula [23]:

PNI = serum albumin (g/L) + 5 × total lymphocyte count (10^9^/L)

The CONUT score was calculated according to the original validated scoring system [24] using serum albumin concentration, total lymphocyte count, and total cholesterol level. Albumin contributes 0–6 points, lymphocyte count 0–3 points, and total cholesterol 0–3 points, yielding a total score ranging from 0 to 12. Higher CONUT scores indicate worse nutritional status. Iron-related laboratory parameters, including serum iron, ferritin, and transferrin saturation, were also collected for descriptive analyses.

### 2.5. Echocardiographic Assessment

Transthoracic echocardiographic (TTE) examinations performed during the index hospitalization were reviewed retrospectively through the institutional reporting system. Echocardiographic studies were not reanalyzed specifically for the purpose of this investigation; instead, measurements reported during routine clinical practice by board-certified cardiologists were used.

LVEF was determined using the modified Simpson biplane method according to contemporary guideline recommendations [25]. Patients with LVEF < 50% were eligible for inclusion in the final cohort, whereas those with preserved ejection fraction (LVEF ≥ 50%) were excluded.

### 2.6. Statistical Analysis

Statistical analyses were performed using IBM SPSS Statistics version 29.0 (IBM Corp., Armonk, NY, USA) and R version 4.6.1 (R Foundation for Statistical Computing, Vienna, Austria).

The distribution of continuous variables was assessed using the Shapiro–Wilk test and visual inspection of histograms. Normally distributed variables are presented as mean ± standard deviation (SD), whereas non-normally distributed variables are expressed as median and interquartile range (IQR). Categorical variables are reported as frequencies and percentages.

Comparisons between survivors and non-survivors were performed using the independent-samples *t*-test for normally distributed continuous variables and the Mann–Whitney U test for non-normally distributed variables. Categorical variables were compared using the χ^2^ test or Fisher’s exact test, as appropriate.

Receiver operating characteristic (ROC) curve analysis was performed to evaluate the discriminatory performance of the TPMA, PNI, and CONUT scores for predicting all-cause mortality. Areas under the curves (AUCs) with corresponding 95% confidence intervals (CIs) were calculated, and optimal cutoff values were determined using the Youden index.

Kaplan–Meier survival curves were generated to assess the association between TPMA, PNI, and long-term survival. Differences between survival curves were evaluated using the log-rank test.

Correlations between TPMA and nutritional indices (PNI and CONUT) were assessed using Spearman rank correlation analysis. The association between clinical variables and all-cause mortality was evaluated using Cox proportional hazards regression analysis. Variables demonstrating a *p*-value < 0.10 in univariable analyses, together with clinically relevant covariates, were considered for multivariable modeling. Given the known influence of sex on skeletal muscle area, the robustness of the association between low TPMA and mortality was additionally examined in a Cox model adjusted for sex, age, LVEF, and eGFR, and a sex-by-TPMA interaction term was tested to assess potential effect modification by sex. Hazard ratios (HRs) and corresponding 95% confidence intervals were reported. All statistical tests were two-sided, and a *p*-value < 0.05 was considered statistically significant.

## 3. Results

### 3.1. Baseline Characteristics

A total of 138 patients with decompensated HF and an LVEF below 50% (HFmrEF/HFrEF) were included in the study. Over a median follow-up of 34.4 months (IQR 12.6–60.9 months), 87 patients (63.0%) died and 51 (37.0%) survived. Baseline clinical, laboratory, echocardiographic, nutritional, and muscle-related characteristics are presented in Table 1. Non-survivors had lower hemoglobin levels (11.8 IQR 10.5–13.0 vs. 12.4 IQR 11.3–13.3 g/dL, *p* = 0.035), lower serum albumin concentrations (38.3 ± 4.1 vs. 40.5 ± 4.2 g/L, *p* = 0.004), and significantly higher BNP levels (1238 IQR 754–2099 vs. 518 IQR 235–1202 pg/mL, *p* < 0.001) compared with survivors. Differences were also observed in nutritional and muscle-related measures. Non-survivors had lower TPMA values (2589 IQR 2071–3060 vs. 3012 IQR 2410–3943 mm^2^, *p* = 0.002) and lower PNI scores (46.0 ± 5.5 vs. 50.2 ± 7.2, *p* < 0.001). In contrast, CONUT score did not differ significantly between survivors and non-survivors (2 IQR 1–3 vs. 2 IQR 1–3, *p* = 0.225) (Table 1). During the index hospitalization, 104 patients (75.4%) received an ACEi, ARB, or ARNI, 121 (87.7%) received a beta-blocker, 77 (55.8%) received an MRA, and 13 (9.4%) received an SGLT2 inhibitor. Medication use according to survival status is presented in Table 1.

### 3.2. Discriminatory Performance of TPMA, PNI, and CONUT

**Figure 3:**
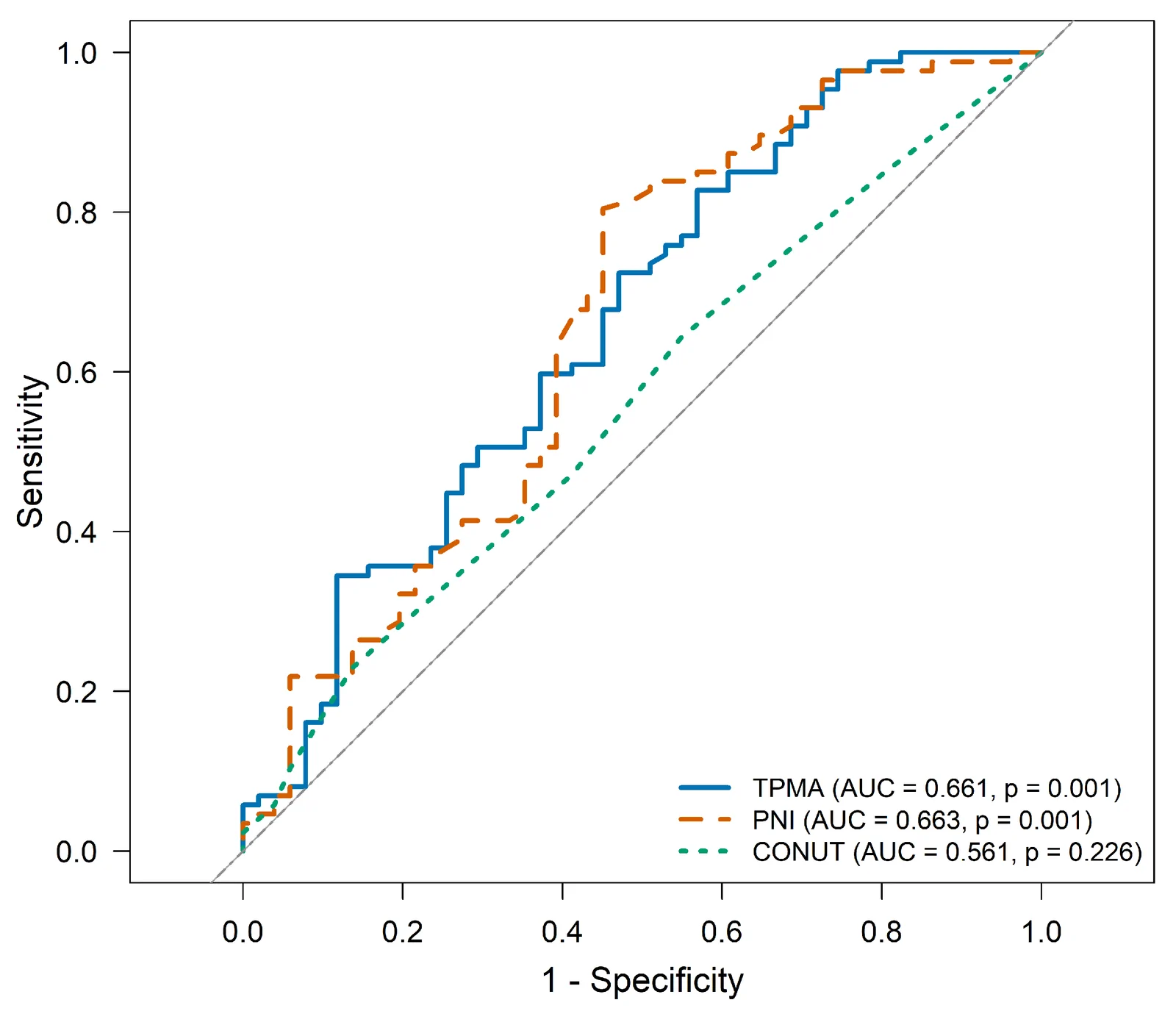
Receiver operating characteristic curves of total pectoral muscle area (TPMA), Prognostic Nutritional Index (PNI), and Controlling Nutritional Status (CONUT) scores for predicting all-cause mortality. The diagonal reference line represents no discriminatory ability (AUC = 0.5). AUC, area under the receiver operating characteristic curve.

Receiver operating characteristic analysis demonstrated significant discriminatory ability for both TPMA and PNI in predicting all-cause mortality (Table 2 and Figure 3). TPMA yielded an AUC of 0.661 (95% CI 0.565–0.758, *p* = 0.001), while PNI demonstrated a similar performance with an AUC of 0.663 (95% CI 0.564–0.761, *p* = 0.001). In contrast, the CONUT score showed limited discriminatory performance (AUC 0.561, 95% CI 0.462–0.659, *p* = 0.226). The optimal ROC-derived cutoff values were 3258 mm^2^ for TPMA and 50.5 for PNI. For clinical applicability, TPMA and PNI were subsequently dichotomized using thresholds of 3000 mm^2^ and 50, respectively.

### 3.3. Survival Analyses

**Figure 4:**
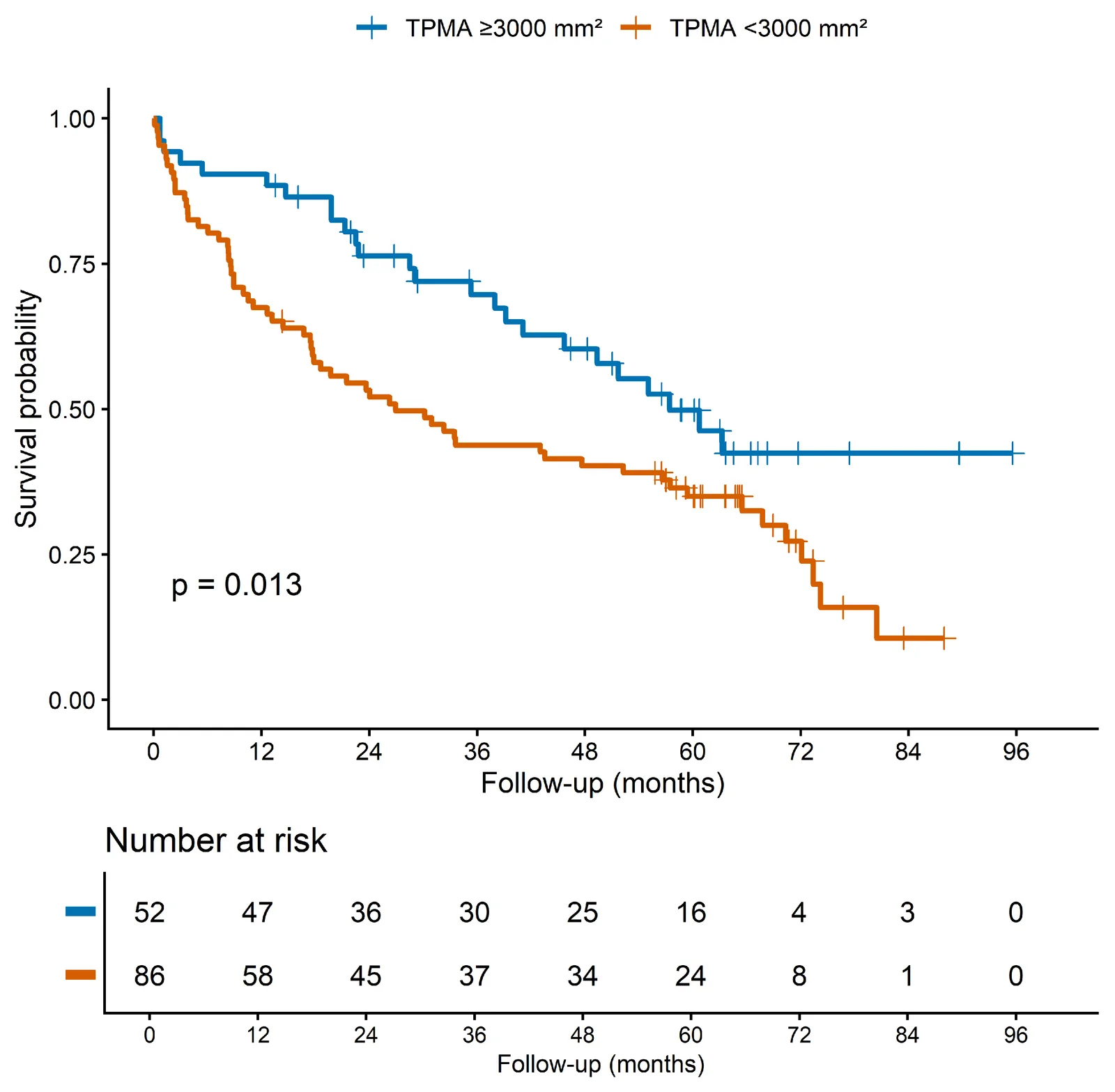
Kaplan–Meier survival curves according to total pectoral muscle area (TPMA). Patients with TPMA < 3000 mm^2^ had significantly lower survival than those with TPMA ≥ 3000 mm^2^ (log-rank *p* = 0.013). The number of patients at risk is shown below the survival curves.

**Figure 5:**
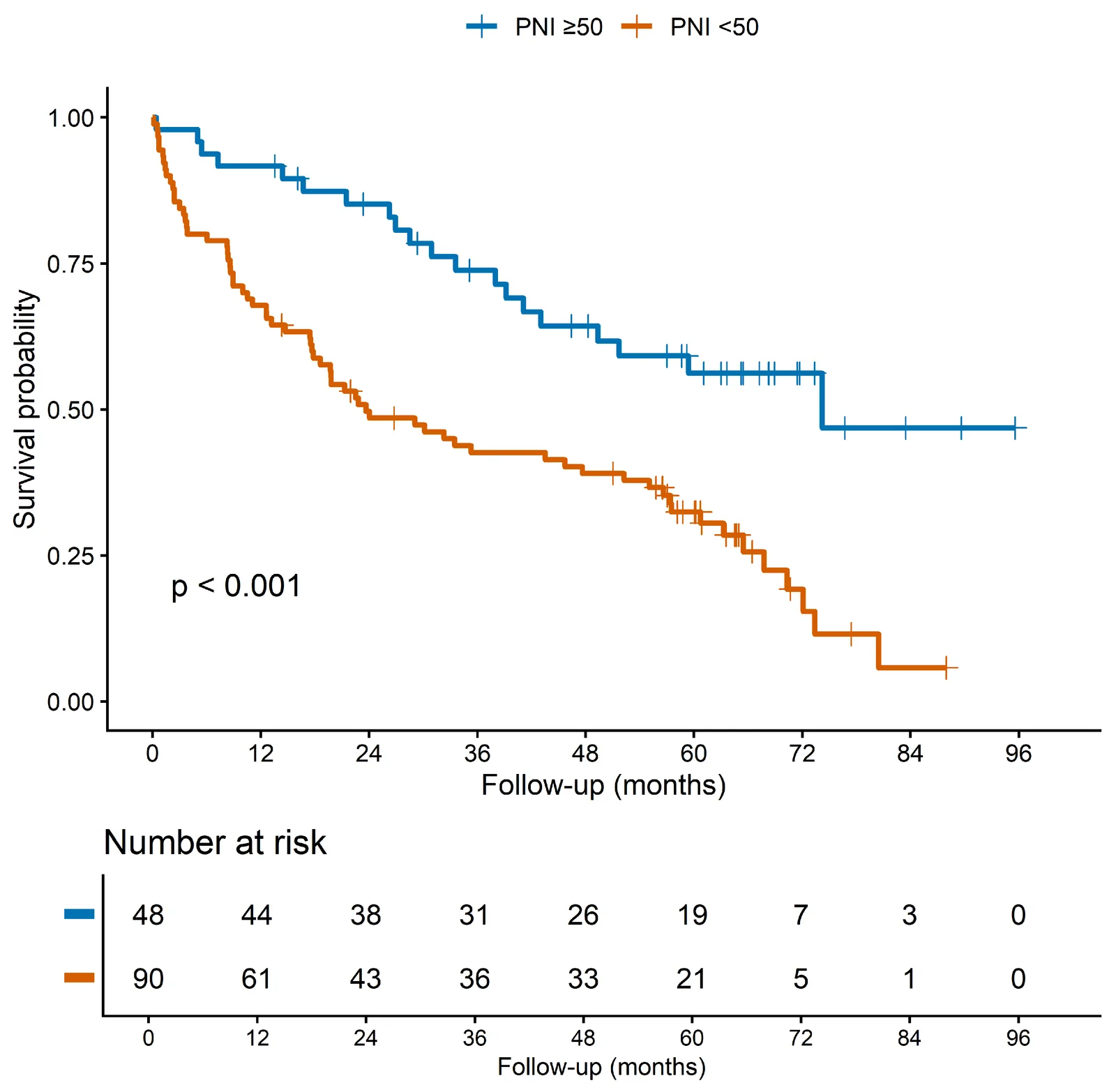
Kaplan–Meier survival curves according to Prognostic Nutritional Index (PNI). Patients with PNI < 50 had significantly lower survival than those with PNI ≥ 50 (log-rank *p* < 0.001). The number of patients at risk is shown below the survival curves.

Kaplan–Meier survival analysis demonstrated significantly lower survival among patients with TPMA < 3000 mm^2^ compared with those with TPMA ≥ 3000 mm^2^ (log-rank *p* = 0.013; Figure 4). Similarly, patients with PNI < 50 had significantly reduced survival compared with patients with PNI ≥ 50 (log-rank *p* < 0.001; Figure 5).

For TPMA, estimated survival rates at 1, 3, and 5 years were 66.3%, 48.5%, and 35.0% in the low-TPMA group compared with 88.5%, 69.7%, and 46.3% in patients with TPMA ≥ 3000 mm^2^. For PNI, mean survival was 36.3 months (95% CI 29.9–42.7) in patients with PNI < 50 and 64.8 months (95% CI 54.5–75.1) in those with PNI ≥ 50.

### 3.4. Correlation Analyses

TPMA demonstrated a modest positive correlation with PNI (Spearman’s rho = 0.325, 95% CI 0.162–0.471, *p* < 0.001) and a weaker inverse correlation with the CONUT score (Spearman’s rho = −0.213, 95% CI −0.371 to −0.042, *p* = 0.012).

### 3.5. Predictors of All-Cause Mortality

In univariable Cox regression analyses, lower hemoglobin, higher BNP levels, lower sodium and chloride concentrations, lower albumin levels, lower lymphocyte count, lower total cholesterol levels, lower TPMA, higher CONUT score, lower PNI, and low TPMA (<3000 mm^2^) were significantly associated with all-cause mortality (Table 3).

In the multivariable model, BNP, PNI, and low TPMA remained independently associated with mortality. Each 100 pg/mL increase in BNP was associated with a 2.4% increase in mortality risk (HR 1.024, 95% CI 1.005–1.044, *p* = 0.014). Low TPMA (<3000 mm^2^) was independently associated with nearly a two-fold higher risk of mortality (HR 1.911, 95% CI 1.118–3.266, *p* = 0.018). Higher PNI remained protective, with each one-point increase associated with a 4.4% reduction in mortality risk (HR 0.956, 95% CI 0.919–0.994, *p* = 0.024) (Table 3). Descriptively, mean TPMA was 2315.7 ± 617.0 mm^2^ in women and 3144.5 ± 1020.6 mm^2^ in men. Given the known sex-related differences in skeletal muscle area, the robustness of the association between low TPMA and mortality was further examined in a model adjusted for sex, age, LVEF, and eGFR. Low TPMA remained independently associated with all-cause mortality (HR 2.296, 95% CI 1.358–3.881; *p* = 0.002). In addition, no significant interaction was observed between sex and low TPMA with respect to all-cause mortality (*p* for interaction = 0.331).

## 4. Discussion

The present study simultaneously evaluated thoracic CT-derived pectoral muscle area and established nutritional indices (PNI and CONUT) in patients hospitalized with decompensated HFmrEF/HFrEF, allowing assessment of their interrelationship and prognostic associations. The principal findings were as follows: (i) reduced TPMA was independently associated with long-term all-cause mortality; (ii) lower PNI was independently associated with mortality; (iii) although CONUT was associated with mortality in univariable analyses, this association did not persist after multivariable adjustment; and (iv) TPMA demonstrated a modest but significant correlation with PNI, indicating that imaging-derived muscle reserve and laboratory-based nutritional status may capture related but partly distinct aspects of risk.

Loss of skeletal muscle mass is increasingly recognized as an important marker of adverse outcomes in HF [2,4]. HF-associated muscle wasting is multifactorial and has been linked to chronic inflammation, neurohormonal and hormonal alterations, metabolic dysregulation, anabolic resistance, malnutrition, and reduced physical activity [3,11]. These changes may contribute to frailty, impaired exercise capacity, and poorer clinical outcomes [3,11]. A recent systematic review and meta-analysis demonstrated that reduced skeletal muscle mass is associated with increased mortality in patients with HF, underscoring the prognostic relevance of skeletal muscle depletion in this population [4]. Our findings are concordant with these observations and further support the prognostic significance of reduced skeletal muscle reserve in hospitalized patients with HF.

Several studies have evaluated imaging-derived skeletal muscle measurements in HF populations. Mirzai et al. demonstrated that thoracic CT-derived skeletal muscle measurements correlate with conventional abdominopelvic CT-based assessments, supporting their potential use for opportunistic assessment of muscle quantity and quality in patients with HF [7]. Huang et al. reported that low skeletal muscle mass determined by chest CT was independently associated with both all-cause and cardiovascular mortality in patients hospitalized with acute HF [8]. Similarly, Reyes-Paz et al. found an association between reduced pectoral skeletal muscle area and increased in-hospital mortality in patients with acute HF [9]. In patients with advanced HF undergoing LVAD implantation, Teigen et al. reported that pectoralis muscle quantity and attenuation were associated with post-LVAD mortality [10]. Collectively, these findings suggest that thoracic imaging-derived muscle measurements may provide prognostic information across different HF populations. In our cohort, the association between low TPMA and mortality remained significant after adjustment for PNI and other clinical variables, suggesting that CT-derived muscle quantity may provide information that is not fully captured by laboratory-based nutritional assessment.

Malnutrition is a common and frequently underrecognized component of HF. Its development is multifactorial and may involve inflammation, intestinal congestion, neurohormonal activation, and reduced nutritional intake, with impaired nutritional status being associated with adverse clinical outcomes [11]. Among laboratory-based nutritional indices, PNI has been investigated as a prognostic marker in HF. Gu et al. reported that lower PNI values were associated with increased mortality in patients with acute decompensated HF [14], while Candeloro et al. found that low PNI was independently associated with both short- and long-term mortality among elderly patients hospitalized with acute HF [15]. Associations between lower PNI and adverse outcomes have also been reported in patients with cardiac pacemakers [16]. In our cohort, lower PNI remained independently associated with mortality after multivariable adjustment, consistent with previous evidence supporting its potential prognostic relevance in HF.

A notable finding of the present study was the differential performance of PNI and CONUT. Although higher CONUT scores were associated with mortality in univariable analysis, this association was attenuated after multivariable adjustment. This finding differs from recent systematic reviews and meta-analyses reporting associations between higher CONUT scores and adverse outcomes in HF populations [17,18]. Several factors may contribute to this discrepancy. The prognostic information captured by CONUT may partly overlap with that of its individual components and other clinical or laboratory variables included in multivariable models. In addition, differences in patient characteristics, HF severity, study design, and covariate adjustment across studies may influence the observed prognostic performance of nutritional indices. Our findings therefore suggest that the prognostic associations of different nutritional indices may vary according to the population studied and the components incorporated into each score.

A relevant observation of the present study was that TPMA and PNI remained independently associated with mortality despite a modest positive correlation between them. Skeletal muscle wasting and nutritional impairment share several biological pathways, including inflammation, anabolic resistance, hormonal alterations, and reduced nutritional intake [3,11]. Nevertheless, the persistence of both variables in multivariable analyses suggests that they may capture partly distinct aspects of patient vulnerability. TPMA primarily reflects skeletal muscle quantity and body composition, whereas PNI integrates laboratory markers related to nutritional, inflammatory, and immunological status. The modest correlation observed between TPMA and PNI further suggests that these measures are related but not interchangeable. Accordingly, their concurrent assessment may provide complementary information; however, the incremental prognostic value of combining TPMA and PNI requires confirmation in dedicated predictive analyses and external cohorts.

From a practical perspective, both TPMA and PNI can be derived from data that may already be available during routine clinical care. Thoracic CT examinations obtained for clinical indications can also be used for opportunistic assessment of pectoral muscle area without additional imaging or radiation exposure [3,7]. Likewise, PNI can be calculated from routinely measured laboratory parameters. These characteristics suggest that TPMA and PNI may be feasible adjunctive markers for risk assessment in hospitalized patients with HF, although their incremental clinical value requires further prospective evaluation.

### Limitations

First, this was a retrospective, single-center study and was therefore susceptible to residual confounding and selection bias. In particular, inclusion required thoracic CT imaging during the index hospitalization. Because CT examinations were obtained for clinical indications rather than according to a predefined study protocol, patients undergoing thoracic CT may represent a clinically selected subgroup of the overall hospitalized HF population. Furthermore, the specific indications for CT imaging could not be systematically categorized for all patients, and the timing and acquisition protocols were not fully standardized.

Second, TPMA was analyzed as an absolute measurement and was not indexed to height, body surface area, or other anthropometric measures because reliable anthropometric data were not consistently available in this retrospective cohort. As pectoral muscle area is influenced by body size and sex, the lack of anthropometric normalization should be considered when interpreting the absolute TPMA values and the 3000 mm^2^ threshold identified in this cohort. Although low TPMA remained independently associated with mortality after adjustment for sex and no significant sex-by-TPMA interaction was observed, the absence of a significant interaction should be interpreted cautiously given the limited sample size. Both height and weight were available in only 20 of 138 patients (14.5%), precluding reliable normalization of TPMA to height, body surface area, or BMI.

Third, TPMA represents skeletal muscle quantity rather than muscle quality or function. CT-derived muscle area does not capture muscle attenuation, intramuscular adipose infiltration, or myosteatosis, which may impair muscle quality and strength despite relatively preserved muscle mass. In addition, muscle strength and physical performance were not assessed using functional measures such as handgrip strength, chair-stand testing, or gait speed. Therefore, low TPMA should not be interpreted as equivalent to clinically defined sarcopenia.

Fourth, PNI and CONUT were calculated from single baseline laboratory measurements obtained during the index hospitalization. Because serum albumin, lymphocyte count, and total cholesterol may be influenced by acute inflammation, hemodilution, congestion, and disease severity during decompensated HF, these indices may not fully reflect patients’ chronic nutritional status. Furthermore, serial changes in nutritional status were not assessed.

Fifth, interobserver and intraobserver reproducibility analyses were not performed, which limits the assessment of measurement variability and the reproducibility of TPMA measurements.

Sixth, the study period extended from 2017 to 2025, during which guideline-directed medical therapy for HF evolved substantially. Consequently, treatment patterns were heterogeneous across the cohort, particularly for newer therapies such as sodium–glucose cotransporter 2 inhibitors, and residual treatment-era confounding cannot be excluded.

Seventh, the relatively modest sample size may limit the precision and stability of the multivariable estimates and may increase the risk of model overfitting. In addition, the TPMA and PNI thresholds were derived and evaluated within the same cohort, which may have resulted in optimistic estimates of their prognostic performance. External validation of the identified thresholds and prognostic associations is therefore required.

Finally, the findings are limited to hospitalized patients with HFmrEF and HFrEF and may not be directly generalizable to ambulatory HF populations, patients with preserved ejection fraction, or other healthcare settings.

## 5. Conclusions

In patients hospitalized with decompensated HF and a left ventricular ejection fraction below 50%, both reduced TPMA and lower PNI were independently associated with increased long-term mortality. While CONUT demonstrated prognostic value in univariable analyses, it did not retain independent significance after adjustment for clinical variables. TPMA and PNI were modestly correlated and may capture partly distinct aspects of systemic impairment. Further studies are needed to determine whether their combined assessment provides incremental prognostic value beyond established risk markers.

## Figures and Tables

**Table 1:** Baseline clinical, laboratory, echocardiographic, nutritional and body composition characteristics according to all-cause mortality.

Variable	Survivors (*n* = 51)	Non-Survivors (*n* = 87)	*p*-Value
Age, years	65 (58–75)	66 (61–75.5)	0.741
Female sex, *n* (%)	16 (31.4)	33 (37.9)	0.437
Hypertension, *n* (%)	40 (78.4)	65 (74.7)	0.621
Diabetes mellitus, *n* (%)	27 (52.9)	41 (47.1)	0.510
Coronary artery disease, *n* (%)	31 (60.8)	63 (72.4)	0.157
Current smoking, *n* (%)	25 (49.0)	48 (55.2)	0.485
Hemoglobin, g/dL	12.4 (11.3–13.3)	11.8 (10.5–13.0)	**0.035**
Creatinine, mg/dL	1.05 (0.82–1.32)	1.07 (0.86–1.32)	0.637
eGFR, mL/min/1.73 m^2^	69 (52–88)	65 (49–82)	0.305
Lymphocyte count, ×10^9^/L	1.69 (1.13–2.63)	1.49 (1.15–1.94)	0.054
Platelet count, ×10^9^/L	222 (192–271)	216 (178–276)	0.490
CRP, mg/L	5.2 (2.1–9.0)	5.8 (3.0–11.4)	0.355
Albumin, g/L	40.5 ± 4.2	38.3 ± 4.1	**0.004**
Total cholesterol, mg/dL	169 (134–194)	151 (127–192)	0.293
HDL cholesterol, mg/dL	40 (30–45)	37 (30–46)	0.593
LDL cholesterol, mg/dL	104 (77–124)	92 (74–119)	0.396
Triglycerides, mg/dL	109 (84–171)	103 (79–139)	0.316
Sodium, mmol/L	138 (136–141)	137 (134–139)	0.063
Chloride, mmol/L	104 (101–107)	103 (98–106)	0.068
Potassium, mmol/L	4.23 ± 0.51	4.40 ± 0.62	0.120
BNP, pg/mL	518 (235–1202)	1238 (754–2099)	**<0.001**
Ferritin, ng/mL	72 (45–141)	55 (36–103)	0.133
Serum iron, µg/dL	45 (37–66)	40 (28–59)	0.121
TSAT, %	13.7 (10.4–19.4)	12.1 (8.1–19.9)	0.274
Left ventricular ejection fraction, %	35 (25–44.5)	33 (25–40)	0.241
Heart failure pharmacotherapy			
ACEi/ARB/ARNI, *n* (%)	40 (78.4)	64 (73.6)	0.522
Beta-blocker, *n* (%)	45 (88.2)	76 (87.4)	0.879
MRA, *n* (%)	27 (52.9)	50 (57.5)	0.605
SGLT2 inhibitor, *n* (%)	9 (17.6)	4 (4.6)	**0.016**
TPMA, mm^2^	3012 (2410–3943)	2589 (2071–3060)	**0.002**
PNI	50.2 ± 7.2	46.0 ± 5.5	**<0.001**
CONUT score	2 (1–3)	2 (1–3)	0.225

Abbreviations: ACEi, angiotensin-converting enzyme inhibitor; ARB, angiotensin receptor blocker; ARNI, angiotensin receptor–neprilysin inhibitor; BNP, B-type natriuretic peptide; CONUT, Controlling Nutritional Status; CRP, C-reactive protein; eGFR, estimated glomerular filtration rate; HDL, high-density lipoprotein cholesterol; LDL, low-density lipoprotein cholesterol; MRA, mineralocorticoid receptor antagonist; *n*, number of patients; PNI, prognostic nutritional index; SGLT2, sodium–glucose cotransporter 2; TPMA, total pectoral muscle area; TSAT, transferrin saturation. Data are presented as mean ± standard deviation, median (interquartile range), or *n* (%), as appropriate. Bold *p*-values indicate statistical significance (*p* < 0.05).

**Table 2:** Discriminatory performance of TPMA, PNI, and CONUT scores for all-cause mortality.

Variable	AUC (95% CI)	*p*-Value
TPMA	0.661 (0.565–0.758)	0.001
PNI	0.663 (0.564–0.761)	0.001
CONUT	0.561 (0.462–0.659)	0.226

**Abbreviations:** AUC, area under the receiver operating characteristic curve; CI, confidence interval; CONUT, Controlling Nutritional Status score; PNI, prognostic nutritional index; TPMA, total pectoral muscle area.

**Table 3:** Univariable and multivariable Cox regression analyses of clinical, nutritional, and body composition parameters associated with all-cause mortality.

Variable	Univariable Analysis				Multivariable Analysis			
	HR	95% CI		*p*-Value	HR	95% CI		*p*-Value
		Lower	Upper			Lower	Upper	
Age, years	0.996	0.979	1.012	0.603	0.989	0.971	1.007	0.244
Male sex (vs. female)	1.033	0.669	1.595	0.884	1.554	0.934	2.588	0.090
Hemoglobin, g/dL	0.853	0.741	0.983	**0.028**	0.913	0.779	1.071	0.263
Left ventricular ejection fraction, %	0.981	0.959	1.003	**0.089**	0.996	0.970	1.023	0.774
BNP, per 100 pg/mL	1.036	1.019	1.054	**<0.001**	1.024	1.005	1.044	**0.014**
Low TPMA, <3000 mm^2^	1.793	1.126	2.856	**0.014**	1.911	1.118	3.266	**0.018**
PNI	0.929	0.897	0.963	**<0.001**	0.956	0.919	0.994	**0.024**
Atrial fibrillation	1.555	0.996	2.428	**0.052**	1.460	0.920	2.317	0.109
Hypertension	0.754	0.463	1.228	0.257				
Diabetes mellitus	0.883	0.578	1.348	0.564				
Coronary artery disease	1.295	0.809	2.072	0.282				
Current smoking	1.314	0.860	2.006	0.206				
eGFR, mL/min/1.73 m^2^	0.998	0.988	1.008	0.680				
Sodium, mmol/L	0.946	0.903	0.992	**0.021**				
Chloride, mmol/L	0.964	0.931	1.000	**0.047**				
Albumin, g/L	0.911	0.863	0.962	**<0.001**				
Lymphocyte count, ×10^9^/L	0.612	0.451	0.830	**0.002**				
Total cholesterol, mg/dL	0.995	0.990	1.000	**0.035**				
LDL cholesterol, mg/dL	0.994	0.989	1.000	**0.070**				
HDL cholesterol, mg/dL	0.988	0.970	1.006	0.187				
Triglycerides, mg/dL	0.997	0.994	1.001	**0.098**				
TPMA, per 100 mm^2^	0.963	0.940	0.987	**0.002**				
CONUT score	1.196	1.051	1.361	**0.007**				

Abbreviations: BNP; B-type natriuretic peptide; CI, confidence interval; CONUT, Controlling Nutritional Status; eGFR, estimated glomerular filtration rate; HDL, high-density lipoprotein cholesterol; HR, hazard ratio; LDL, low-density lipoprotein cholesterol; PNI, Prognostic Nutritional Index; TPMA, total pectoral muscle area. Bold *p*-values indicate variables meeting the prespecified threshold for consideration in the multivariable model (*p* < 0.10) in univariable analysis and statistically significant associations (*p* < 0.05) in multivariable analysis.

## Data Availability

The datasets generated and/or analyzed during the current study are not publicly available due to ethical and privacy restrictions but are available from the corresponding author on reasonable request and with permission of the Institutional Ethics Committee.

## Abbreviations

AF	Atrial Fibrillation
AUC	Area Under the Curve
BNP	B-Type Natriuretic Peptide
CAD	Coronary Artery Disease
CI	Confidence Interval
CONUT	Controlling Nutritional Status
CRP	C-Reactive Protein
CT	Computed Tomography
DICOM	Digital Imaging and Communications in Medicine
DM	Diabetes Mellitus
eGFR	Estimated Glomerular Filtration Rate
HF	Heart Failure
HfmrEF	Heart Failure with Mildly Reduced Ejection Fraction
HfrEF	Heart Failure with Reduced Ejection Fraction
HfpEF	Heart Failure with Preserved Ejection Fraction
HDL	High-Density Lipoprotein
HR	Hazard Ratio
HT	Hypertension
ICD	International Classification of Diseases
IQR	Interquartile Range
LDL	Low-Density Lipoprotein
LVEF	Left Ventricular Ejection Fraction
LVAD	Left Ventricular Assist Device
PNI	Prognostic Nutritional Index
ROC	Receiver Operating Characteristic
SD	Standard Deviation
TPMA	Total Pectoral Muscle Area
TSAT	Transferrin Saturation
TTE	Transthoracic Echocardiography

## References

[1] McDonagh T.A., Metra M., Adamo M., Gardner R.S., Baumbach A., Böhm M., Burri H., Butler J., Čelutkienė J., Chioncel O. (2023). 2023 focused update of the 2021 ESC Guidelines for the diagnosis and treatment of acute and chronic heart failure. Eur. Heart J..

[2] Cai X., Liu M., Qin P., Tang S., He L., Lei J., Zhou Y., Xu Z., Guo Y., Feng C. (2025). Skeletal muscle mass and mortality in heart failure: Mediation role of systemic immune-inflammatory index. JACC Adv..

[3] Mirzai S., Eck B.L., Chen P.H., Estep J.D., Tang W.H.W. (2022). Current approach to the diagnosis of sarcopenia in heart failure: A narrative review on the role of clinical and imaging assessments. Circ. Heart Fail..

[4] Mendonça D.D., da Silva W.V.R., Souza G.C., Rados D.V., Biolo A. (2025). Body composition and survival in patients with heart failure: A systematic review and meta-analysis. JACC Heart Fail..

[5] Shi K., Zhang G., Fu H., Li X.-M., Yu S.-Q., Shi R., Yan W.-F., Qian W.-L., Xu H.-Y., Li Y. (2024). Reduced thoracic skeletal muscle size is associated with adverse outcomes in diabetes patients with heart failure and reduced ejection fraction: Quantitative analysis of sarcopenia by using cardiac MRI. Cardiovasc. Diabetol..

[6] Cruz-Jentoft A.J., Bahat G., Bauer J., Boirie Y., Bruyère O., Cederholm T., Cooper C., Landi F., Rolland Y., Sayer A.A. (2019). Sarcopenia: Revised European consensus on definition and diagnosis. Age Ageing.

[7] Mirzai S., Persits I., Martens P., Estep J.D., Tang W.H.W., Chen P.H. (2024). Skeletal muscle quantity and quality evaluation in heart failure: Comparing thoracic versus abdominopelvic CT approaches. Int. J. Cardiovasc. Imaging.

[8] Huang Y., Wang Y., Guo J., Fang P., Kong B., Wang Z., Zuo J. (2025). Impact of chest computed tomography-determined low skeletal muscle mass on the survival of patients with acute heart failure. Front. Cardiovasc. Med..

[9] Reyes-Paz Y., Castillo-Martínez L., García-Sánchez J.R., Bernal-Ceballos F., Villanueva-Juárez J.L., Hernández-Gilsoul T. (2026). Skeletal muscle mass and overhydration are associated with in-hospital mortality in acute heart failure patients. Rev. Assoc. Med. Bras..

[10] Teigen L.M., John R., Kuchnia A.J., Nagel E.M., Earthman C.P., Kealhofer J., Martin C., Cogswell R. (2017). Preoperative pectoralis muscle quantity and attenuation by computed tomography are novel and powerful predictors of mortality after left ventricular assist device implantation. Circ. Heart Fail..

[11] von Haehling S., Ebner N., dos Santos M.R., Springer J., Anker S.D. (2017). Muscle wasting and cachexia in heart failure: Mechanisms and therapies. Nat. Rev. Cardiol..

[12] Kwaśny A., Uchmanowicz I., Juárez-Vela R., Młynarska A., Łokieć K., Czapla M. (2024). Sex-related differences in the impact of nutritional status on in-hospital mortality in heart failure: A retrospective cohort study. Eur. J. Cardiovasc. Nurs..

[13] Kałużna-Oleksy M., Krysztofiak H., Sawczak F., Kukfisz A., Szczechla M., Soloch A., Cierzniak M., Szubarga A., Przytarska K., Dudek M. (2024). Sex differences in the nutritional status and its association with long-term prognosis in patients with heart failure with reduced ejection fraction: A prospective cohort study. Eur. J. Cardiovasc. Nurs..

[14] Gu W., Zhou Y., Hua B., Ma W., Dong L., Shi T., Zou J., Zhu N., Chen L. (2024). Predictive value of the prognostic nutritional index combined with serum chloride levels for the prognosis of patients with acute decompensated heart failure. Heart Vessels.

[15] Candeloro M., Di Nisio M., Balducci M., Genova S., Valeriani E., Pierdomenico S.D., Porreca E. (2020). Prognostic Nutritional Index in elderly patients hospitalized for acute heart failure. ESC Heart Fail..

[16] Zhang J., Yao H., Lu Y., Lian L., Zheng R., Chen C. (2025). Baseline and changes in Prognostic Nutritional Index associate with heart failure hospitalization and all-cause death in patients with cardiac pacemaker. BMC Cardiovasc. Disord..

[17] Yuan Y., Wang S.-P., Guan Y., Yang Q.-Y., Zhong P.-Y., Wang H.-Y. (2025). Association between Controlling Nutritional Status score and the prognosis of patients with heart failure: A systematic review and meta-analysis. Front. Cardiovasc. Med..

[18] Fărcaș D.A., Cerghizan A., Maior R., Mîndrilă A.-C., Tarcea M. (2025). CONUT score as a predictor of mortality risk in acute and chronic heart failure: A meta-analytic review. Nutrients.

[19] Ponikowski P., Voors A.A., Anker S.D., Bueno H., Cleland J.G.F., Coats A.J.S., Falk V., González-Juanatey J.R., Harjola V.-P., Jankowska E.A. (2016). 2016 ESC Guidelines for the diagnosis and treatment of acute and chronic heart failure. Eur. Heart J..

[20] McDonagh T.A., Metra M., Adamo M., Gardner R.S., Baumbach A., Böhm M., Burri H., Butler J., Čelutkienė J., Chioncel O. (2021). 2021 ESC Guidelines for the diagnosis and treatment of acute and chronic heart failure. Eur. Heart J..

[21] McDonald M.-L.N., Diaz A.A., Ross J.C., San Jose Estepar R., Zhou L., Regan E.A., Eckbo E., Muralidhar N., Come C.E., Cho M.H. (2014). Quantitative computed tomography measures of pectoralis muscle area and disease severity in chronic obstructive pulmonary disease: A cross-sectional study. Ann. Am. Thorac. Soc..

[22] Moon S.W., Kim S.Y., Choi J.S., Leem A.Y., Lee S.H., Park M.S., Kim Y.S., Chung K.S. (2021). Thoracic skeletal muscle quantification using computed tomography and prognosis of elderly ICU patients. Sci. Rep..

[23] Onodera T., Goseki N., Kosaki G. (1984). Prognostic Nutritional Index in gastrointestinal surgery of malnourished cancer patients. Nihon Geka Gakkai Zasshi.

[24] Ignacio de Ulíbarri J., González-Madroño A., de Villar N.G.P., González P., González B., Mancha A., Rodríguez F., Fernández G. (2005). CONUT: A tool for controlling nutritional status. First validation in a hospital population. Nutr. Hosp..

[25] Lang R.M., Badano L.P., Mor-Avi V., Afilalo J., Armstrong A., Ernande L., Flachskampf F.A., Foster E., Goldstein S.A., Kuznetsova T. (2015). Recommendations for cardiac chamber quantification by echocardiography in adults: An update from the American Society of Echocardiography and the European Association of Cardiovascular Imaging. J. Am. Soc. Echocardiogr..

